# The effect of gender on food insecurity among HIV-infected people receiving anti-retroviral therapy: A systematic review and meta-analysis

**DOI:** 10.1371/journal.pone.0209903

**Published:** 2019-01-07

**Authors:** Dube Jara Boneya, Ahmed Ali Ahmed, Alemayehu Worku Yalew

**Affiliations:** 1 Department of Public Health, College of Health Sciences, Debre Markos University, Debre Markos, Ethiopia; 2 Department of Preventive Medicine, School of Public Health, College of Health Sciences, Addis Ababa University, Addis Ababa, Ethiopia; National and Kapodistrian University of Athens, GREECE

## Abstract

**Background:**

HIV-infected adults receiving anti-retroviral therapy have a high prevalence of food insecurity in both high- and low-income settings., Women bear an inequitable burden of food insecurity due to lack of control over resources and over household food allocation decision-making. The few studies conducted on the association between food insecurity and gender among HIV-infected adults have inconclusive findings. Therefore, the objective of this systematic review and meta-analysis was to estimate the pooled effect of gender on food insecurity among HIV-infected adults receiving antiretroviral therapy.

**Method:**

We conducted an electronic, web-based search using PubMed, CINAHL, PopLine, MedNar, Embase, Cochrane library, the JBI Library, the Web of Science and Google Scholar. We included studies which reported the association between food insecurity and gender among HIV-infected adults receiving antiretroviral therapy whose age was greater than 18 years. The analysis was conducted using STATA 14 software. A random effects model was used to estimate the pooled effect a 95% confidence interval(CI). Forest plots were used to visualize the presence of heterogeneity. Funnel plots and Egger’s and Begg’s tests were used to check for publication bias.

**Results:**

A total of 776 studies were identified of which seventeen studies were included in the meta-analysis, with a total of 5827 HIV infected adults receiving antiretroviral therapy. We found that the gender of HIV-infected adults receiving anti-retroviral therapy had statistically significant effects on food insecurity. The pooled odds of developing food insecurity among female HIV infected adults receiving anti-retroviral therapy was 53% higher than male HIV infected adults (OR: 1.53, 95% CI: 1.29, 1.83). Our analysis indicate the findings of studies conducted in the high-income countries showed weakest associations between gender and food insecurity than those conducted in low- and middle-income countries.

**Conclusion:**

Our systematic review and meta-analysis showed statistically significant effect of gender on food insecurity among HIV-infected adults receiving anti-retroviral therapy in which odds of food insecurity was higher among female HIV infected adults compared to male HIV-infected adults. These findings suggest that the need to include within food and nutrition interventions for HIV-infected adults receiving antiretroviral treatment, culture- and context-specific gender-based policies to address the sex/gender related vulnerability to food insecurity.

## Background

Human immunodeficiency virus (HIV) infection and the acquired immunodeficiency syndrome (AIDS) remain a critical global health crisis[1]. Globally in 2016, there were 36.7 million people living with HIV of which 48.5% (17.8 million) were women of reproductive age (15-49years)[2]. Of the 4,500 daily new HIV infections among adults aged 15 years and older, 43% are among women and 22% are among young women (15–24years)[2].

HIV-infection and food insecurity are closely associated in both low- and high-income settings. For example, food insecurity defined as having uncertain or limited availability of nutritionally adequate or safe food or the inability to procure food in socially acceptable ways has been identified as the critical contributing factor for poor health outcome among adults living with HIV[3–6]. In addition to harming immunological and clinical outcomes in HIV-infected individuals, food insecurity has also been shown to increase the risk of HIV exposure and infection [7].

Studies show a consistently high prevalence of food insecurity among HIV-infected individuals receiving antiretroviral therapy, with HIV-infected women bear an inequitable burden of food insecurity in both high- and low-income settings [8–15].

Females are biologically, socioeconomically, and socio-culturally at higher risk of HIV infection than males. Gender inequity shape power relations and access to health education, and reduces the power of women to negotiate or practice safer sexual behavior which greatly increases their risk of HIV-infection in many settings [7]. Particularly, females in relationships are often more food-insecure than male partners, as a result of unequal power over economic resources and household food allocation decision-making. In addition, females often serve as caregivers and are therefore constrained in their ability to make further investments in their own skills and education, increasing their susceptibility to food insecurity[16].

Despite the fact females have increased vulnerability to food insecurity, studies conducted on the association between food insecurity and gender among HIV-infected adults receiving antiretroviral therapy across the world (low, middle and high-income countries), have presented controversial and inconclusive evidences. For instance, some studies have found significantly positive effect of gender on food insecurity [13, 14, 17–20], while others failed to demonstrate the effect [3, 9–11, 15, 21–26].

Estimating the pooled effect of gender on food insecurity among HIV-infected adults and identify the reasons for this discrepancy in findings is important for addressing food security-related problems and for implementing focused interventions to address food security in this population. Therefore, the main objective of the current systematic review and meta-analysis is to estimate the pooled effect of gender on food insecurity among HIV—infected adult patients receiving antiretroviral therapy. Our research question is: ‘‘Does gender of HIV-infected adults receiving ant-retroviral therapy determine their food security status?”.

The findings of this analysis will be helpful to policy makers and program planners in the design of appropriate interventions to improve the problems related to the association between food insecurity and gender among HIV infected adults receiving anti-retroviral therapy. The findings would also be useful for clinicians and future researchers in related fields.

## Methods

### Search strategy

This systemic review and meta-analysis was conducted to estimate the effects of gender on food insecurity among HIV-infected adults, receiving antiretroviral therapy. Before beginning our study, we checked for the presence of the existing systematic reviews and meta-analysis on our topic using the DARE database (http://www.library.UCSF.edu) and the Cochrane library to avoid duplication. We also checked the availability of ongoing projects related to the current systematic review and meta-analysis. In addition, we searched the two Trial Registries: ICTRP and Clinical Trials.gov (searched 30 February, 2018). No previous systematic reviews or meta-analyses on the topic were found.

We searched all relevant published studies in the following major databases; *PubMed*, *MEDLINE*, *Google Scholar*, *CINAHL*, *PopLine*, *MedNar*, *Embase*, *the Cochrane library*, *the JBI Library*, *the web of science*, *and African Journals Online*. We also retrieved grey literature using Google and Google Scholar searches. To identify and retrieve additional articles, we also reviewed reference lists of identified studies. Unpublished studies were retrieved from the official websites of international and local organizations and universities.

The search for published studies was restricted by the age of the study participants (HIV-infected adults receiving antiretroviral therapy whose age was greater than 18 years), but was not restricted by time or country. All published and unpublished articles written by the time of our search in February 30, 2018 were included in the systematic review and meta-analysis.

The following search terms were used: food security status, food insecurity, effect of gender, effect of gender, adults living with HIV, patients living with HIV, individuals living with HIV, HIV-infected adults, HIV-infected individuals and antiretroviral therapy separately and/or in combination.

We pre-defined search terms to allow a comprehensive search strategy that included all the important studies. All fields within records and Medical Subject Headings (MeSH terms) were used to help expand the search in advanced PubMed search. The following search strategies were modified for the various databases using the two important Boolean operators and search engines with initial keywords/search terms *1) (“Food security status” OR “food insecurity” AND “effect of sex” OR “effect of gender” AND “adult living with HIV” OR “Patients living with HIV” OR “individual living with HIV” AND “antiretroviral therapy”)*. *2) (“Food insecurity” OR “effect of sex” OR “effect of gender” AND “HIV infected adults” OR “HIV-infected patients” OR “HIV-infected individual” OR “antiretroviral therapy” AND “Ethiopia”)*. The Preferred Reporting Items for Systematic Reviews and Meta-Analyses (PRISMA) guideline was followed during the systematic review (S1 Table) [27].

### Study selection and eligibility criteria

The review included articles that were conducted on the association between food insecurity and gender among HIV-infected people, receiving antiretroviral therapy globally. Participants were HIV-infected adults on antiretroviral therapy whose age was greater than 18 years, regardless of their gender. This review considered both community and institution based studies. The outcomes considered were food insecurity and its association with gender or the effect of gender on food insecurity as measured using the Household Food Insecurity Access Scale (HFIAS)[28, 29]. All study types that were published in the form of journal articles, master’s thesis and dissertation, that were written in English were included in the review. In addition, all studies conducted using cross sectional, case-control and cohort designs were included. We excluded studies conducted on the pediatric age group, studies with the methodological problems, interventional studies, and review articles. Retrieved studies were assessed for inclusion in the final review by reviewing their title, abstract and full-text for their agreement with our eligibility criteria.

### Quality assessment and data extraction

We used reference management software (Endnote version X7.2) to combine database search results and to remove duplicate articles manually. The Joanna Briggs Institute Meta-Analysis of Statistics Assessment and Review Instrument (JBI-MAStARI) was used for critical appraisal of studies (S2 Table) [30]. Data were extracted by two independent reviewers using a standardized data extraction format. The data extraction spreadsheet included primary author name, year of publication, country, study design, sample size, number of the subject outcome, response rate, number male with the outcome, number of females with the outcome, the total number of males and females in the study (S1 Dataset). Disagreement between reviewers in the review process were discussed with review team members until consensus was reached. Discrepancies between two independent reviewers were resolved by involving third reviewer. When access to full-text articles were not available, document authors were contacted once. If no reply was received within a month, the documents were excluded from the study.

### Data analysis and synthesis

The extracted data were categorized and sorted by quality scores and entered into the computer using command window of STATA v.14. Analysis of the data were done using STATA v.14 statistical software. The logarithm and standard error of the odds ratio (OR)for each original study were generated using “generate” command in STATA. Cochrane’s Q statistic (chi-square), I^2^ and p-values were used to check for heterogeneity of the studies’ outcomes. The heterogeneity was considered as low, moderate or high when I^2^ test statistics results were 25%, 50%, and 75% respectively [31]. Forest plots were also used to visualize the presence of heterogeneity. Because we found high level of heterogeneity, we used a random effects model for analysis to estimate the Der Simonian and Laird’s pooled effect. Furthermore, to identify source of heterogeneity, meta regression was conducted and statistically significant results were declared in the presence of heterogeneity. Publication bias was checked using funnel plot of symmetry. Further, the statistical significance of publication bias was checked using Egger and Begg tests [32–34]. A p-value less than 0.05 was used to declare the presence of publication bias. We performed sensitivity analysis using a random effects model to assess the influence of a single study on the overall meta-analysis estimate.

## Results

### Selection and identification of studies

**Fig 1:**
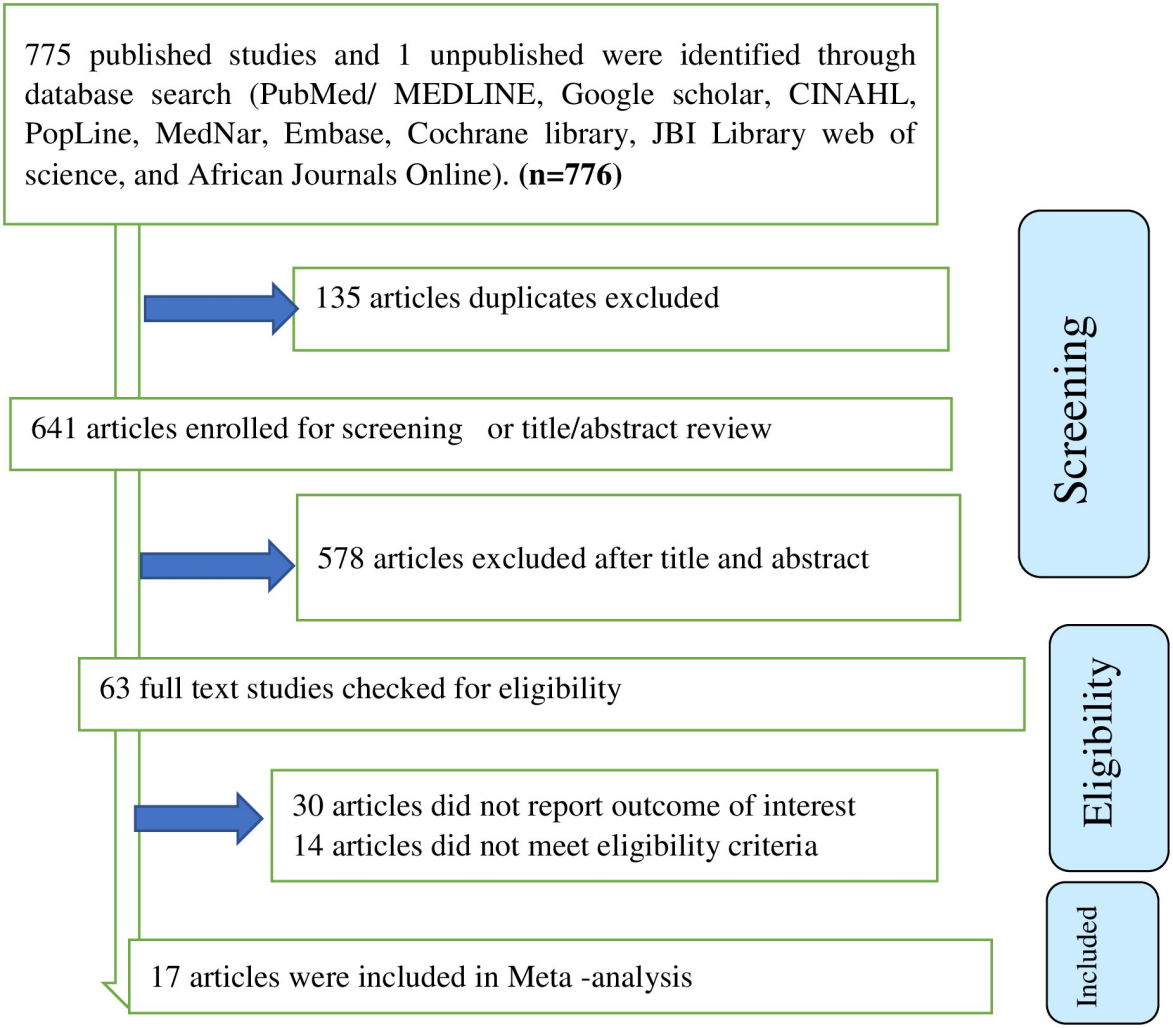
PRISMA flow diagram of included studies in the systematic review and meta-analysis of the effect of gender on food insecurity among HIV-infected people receiving antiretroviral therapy from 2009–2017.

We identified a total of 776 studies (775 published and one unpublished study) that were conducted from 2009 to 2017. Of those identified, 135 duplicate studies were removed and 578 studies were excluded after reviewing of their titles and abstracts. The full text of remaining the 61 studies were assessed for eligibility and for whether they report outcome of interest. Of these, 30 studies were excluded due to lack of outcome of interest and 14 studies were excluded because they failed to meet the eligibility criteria. Of the remaining studies, the 17 that scored- seven and above on the JBI quality appraisal eligibility criteria were included in the final Meta-analysis. The Preferred Reporting Items for Systematic Reviews and Meta-Analyses (PRISMA) flow diagram was used to guide selection process and present the systematic review overview (Fig 1).

### Characteristics of included studies

A total of 17 studies that assessed the association between food insecurity and gender among HIV infected adults receiving anti-retroviral therapy were included in this systematic review and meta-analysis, with a total sample of 5827 individuals living with HIV. Eleven of these studies were cross sectional and six were prospective cohort. The range of minimum and maximum sample size ranged from104 in a study conducted in USA[3]and 796 in a study conducted in Brazil [13].

Of the total 17 included studies, four were conducted in Ethiopia[14, 17, 21, 22], five studies in the United States [3, 15, 19, 20, 25], two in Canada[9, 10], two in India[18, 23], Russia[24], Senegal[11], Uganda[26] and Brazil[13] each had one study. Six studies were conducted in low-income countries[11, 14, 17, 21, 22, 26], four studies in middle-income countries[13, 18, 23, 24] and seven studies in high-income countries[3, 9, 10, 15, 19, 20, 25].

The findings of individual studies were varied and inconclusive with the effects of gender/sex found to be significant in some studies and in- significant other. Of those studies that found significant effects of gender on food insecurity, the strongest positive association was found in the study conducted in Ethiopia[14], with an odds ratio of 4.30 (95% CI: 2.13, 8.68) and the smallest association was found in the study conducted in the United States [20], OR = 1.50 (1.01, 2,.23) (Table 1).

**Table 1:** Characteristics of studies included in the systematic review and meta-analysis on the effect of gender on food insecurity among HIV infected people receiving antiretroviral therapy from 2009–2017.

Study No	Authors	Year	Country	Country income level	Study design	Sample size	Number of subject with outcome	Response rate	Number male with the outcome	Number female with the outcome	Total number of male	Total number of female	OR (95% CI)
1	M. Asnakew[21]	2015	Ethiopia	Low	Cross-sectional	385	260	97.72	88	172	136	249	1.22 (0.78, 1.97)
2	Gedle et al.[17]	2015	Ethiopia	Low	Cross-sectional	338	264	90	91	173	130	208	2.12 (1.26, 3.57)
3	Belijo ZN et al[14]	2017	Ethiopia	Low	Cross-sectional	394	77	100	10	67	134	260	4.30 (2.13, 8.68)
4	Tiyou et al[22]	2012	Ethiopia	Low	Cross-sectional	319	201	100	86	115	144	175	1.29 (0.82, 2.04)
5	Anema et al[9]	2013	Canada	High	Cohort	254	181	100	148	33	211	43	1.40 (0.65, 3.02)
6	Anema et al[10]	2016	Canada	High	Cross-sectional	262	192	100	140	52	191	71	1.00 (0.54, 1.84)
7	Benzekri et al[11]	2015	Senegal	Low	Cross-sectional	109	78	100	12	62	18	91	1.07 (0.36, 3.13)
8	Dasgupta et al[18]	2016	India	Middle	Cross-sectional	173	75	92	33	42	96	77	2.29 (1.24, 4.24)
9	Heylen et al[23]	2015	India	Middle	Cohort	367	58	100	38	20	239	128	0.98 (0.54, 1.77)
10	Idrisov, et al[24]	2017	Russia	Middle	Cohort	310	164	88.32	113	51	220	90	1.24 (0.76, 2.03)
11	Kalichman et al[25]	2013	USA	High	Cross-sectional	197	85	100	66	19	154	43	1.06 (0.53, 2.09)
12	McMahon et al[19]	2011	USA	High	Cohort	592	375	100	239	136	416	176	2.52 (1.68, 3.77)
13	Weiser D. et al[15]	2009	USA	High	Cohort	250	134	100	92	43	174	76	1.16 (0.68, 2.00)
14	Weiser D et al[3]	2009	USA	High	Cross-sectional	104	26	100	15	11	66	40	1.29 (0.52, 3.18)
15	Kalichman et al[20]	2014	USA	High	Cross-sectional	521	321	100	214	107	364	157	1.50 (1.01, 2,.23)
16	Mederios et al[13]	2017	Brazil	Middle	Cross-sectional	796	284	100	143	141	484	312	1.97 (1.46, 2.64)
17	Tsai et al[26]	2012	Uganda	Low	Cohort	456	340	100	93	247	132	324	1.35 (0.86, 2.12)

### The effect of gender on food insecurity among HIV-infected adults receiving anti-retroviral therapy

Our analysis of 17 included studies found significant heterogeneity across studies (I^2^ = 45.5%, p <0.022) which suggested that the use of a fixed effect model might lead to unreliable estimates because those model assume that all heterogeneity can be explained by the covariates. This assumption may create excessive type I errors when there is residual, or unexplained, heterogeneity. To avoid this bias, we used random effects model to estimate the pooled effect of gender on food insecurity among HIV-infected adults in our 17 included studies using an inverse variance method.

**Fig 2:**
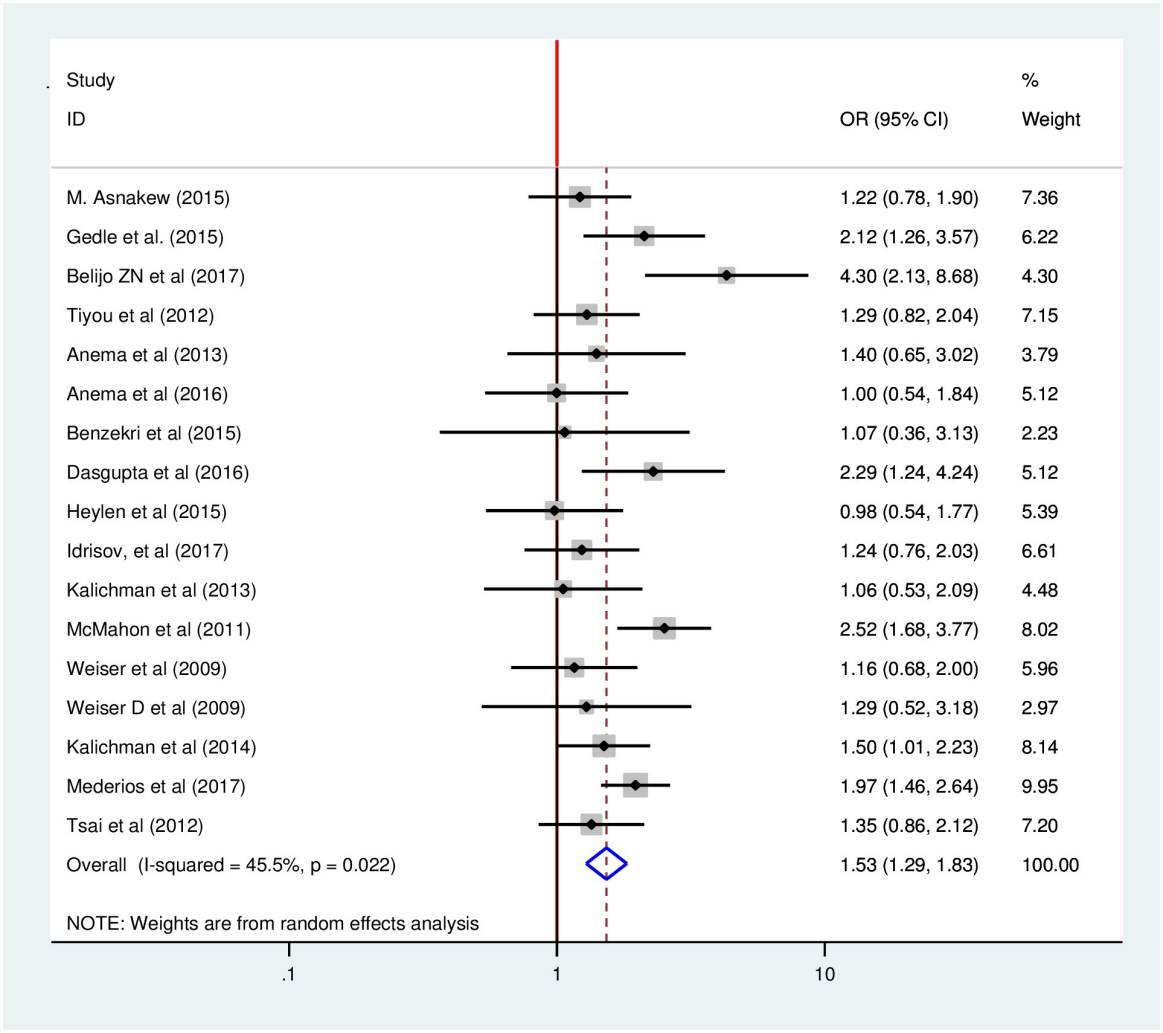
Forest plot of the pooled effect of gender on food insecurity among HIV-infected adults receiving antiretroviral therapy from 2009–2017.

Using these methods, our meta-analysis found that gender of HIV-infected adults receiving anti-retroviral therapy had statistically significant effects on their food security status. The odds of developing food insecurity among female HIV-infected adults receiving anti-retroviral therapy were 53% higher than male HIV-infected adults (OR: 1.53, 95% CI: 1.29, 1.83) (Fig 2).

We further investigated the heterogeneity using different statistical techniques to identify the source of heterogeneity. A meta-regression was performed using publication year, sample size and country income level as covariates and by specifying the method for estimating the between-study variance. None of the three variables were statistically significant for explaining the presence of heterogeneity (Table 2).

**Table 2:** Related factors with heterogeneity of the effects of gender on food insecurity among HIV-infected adults receiving antiretroviral therapy, 2009–2017.

Variables	Coefficients	p-value
Publication Year	0.0284334	0.588
Sample size	0.0008173	0.588
Low income countries	0.0769214	0.751
Middle income countries	-0.1118692	0.731
High income countries	Reference	

**Fig 3:**
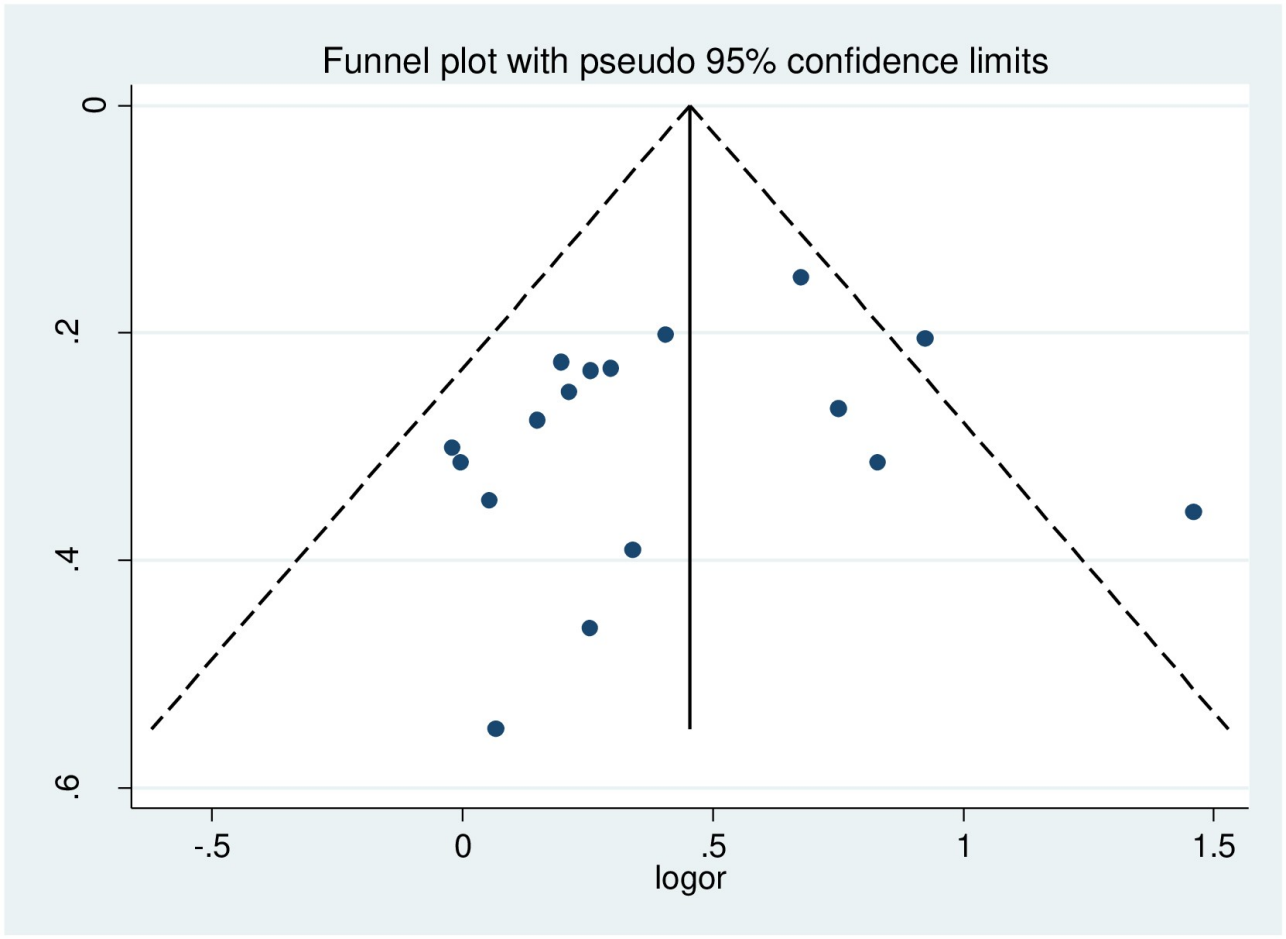
Funnel plots for publication bias of effect of gender on food insecurity among HIV-infected adults receiving antiretroviral therapy from 2009–2017.

The presence of publication bias was assessed using funnel plots and Egger and Begg statistical tests at 5% significant level. There was no statistical evidence of publication bias. The funnel plot was almost symmetry, the Begg and Egger tests were not statistically significant with p-value = 0.484 and p-value = 0.321 respectively (Fig 3).

**Fig 4:**
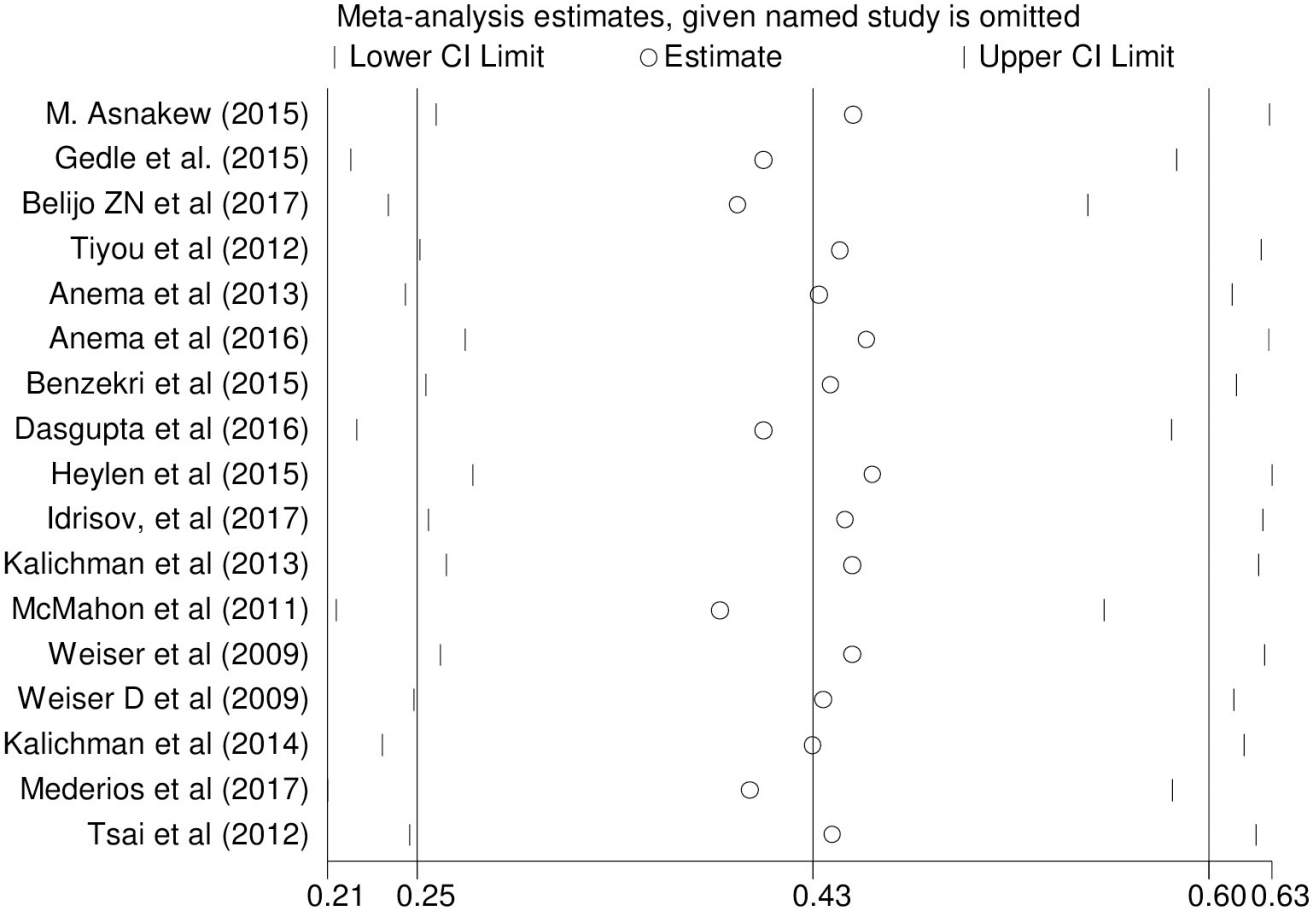
Sensitivity analysis for single study influence of effect of gender on food insecurity among HIV-infected adults receiving antiretroviral therapy from 2009–2017.

To identify the effect of single study on overall meta-analysis estimate, we performed sensitivity analysis using a random effects model. The analysis found no strong evidence for influence of single study (Fig 4).

### Sub-group analysis by study design

**Fig 5:**
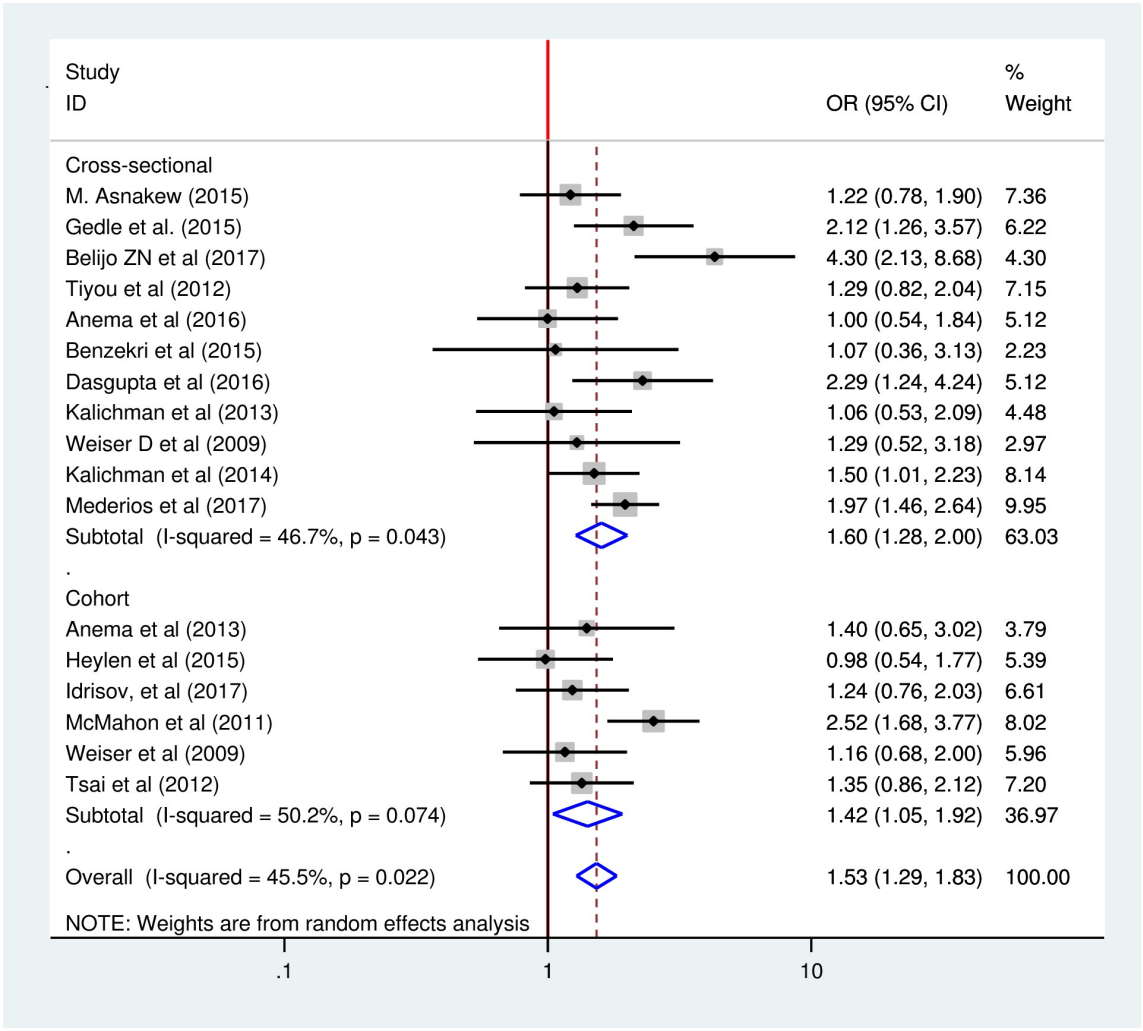
Sub-group analysis of effect of gender on food insecurity among HIV-infected adults receiving antiretroviral therapy by study design from 2009–2017.

We performed sub-group analysis by study design to minimize the potential random variations between studies by comparing the effect of gender on food insecurity of HIV-infected adults. Our sub-group analysis indicated almost consistent significant effect of gender on food insecurity among HIV-infected adults across the study designs. The analysis indicated that cohort studies had weaker associations than cross-sectional studies. The odds of developing food insecurity among female HIV-infected adults was 42% higher compared to male HIV-infected adults in cohort studies while the odds of developing food insecurity among female HIV-infected adults was 60% higher compared to male HIV-infected adults in cross sectional studies, with odds ratios of 1.42 (95% CI: 1.05, 1.92) and 1.60 (95% CI: 1.28, 2.00) respectively (Fig 5).

### Sub-group analysis by country income level

**Fig 6:**
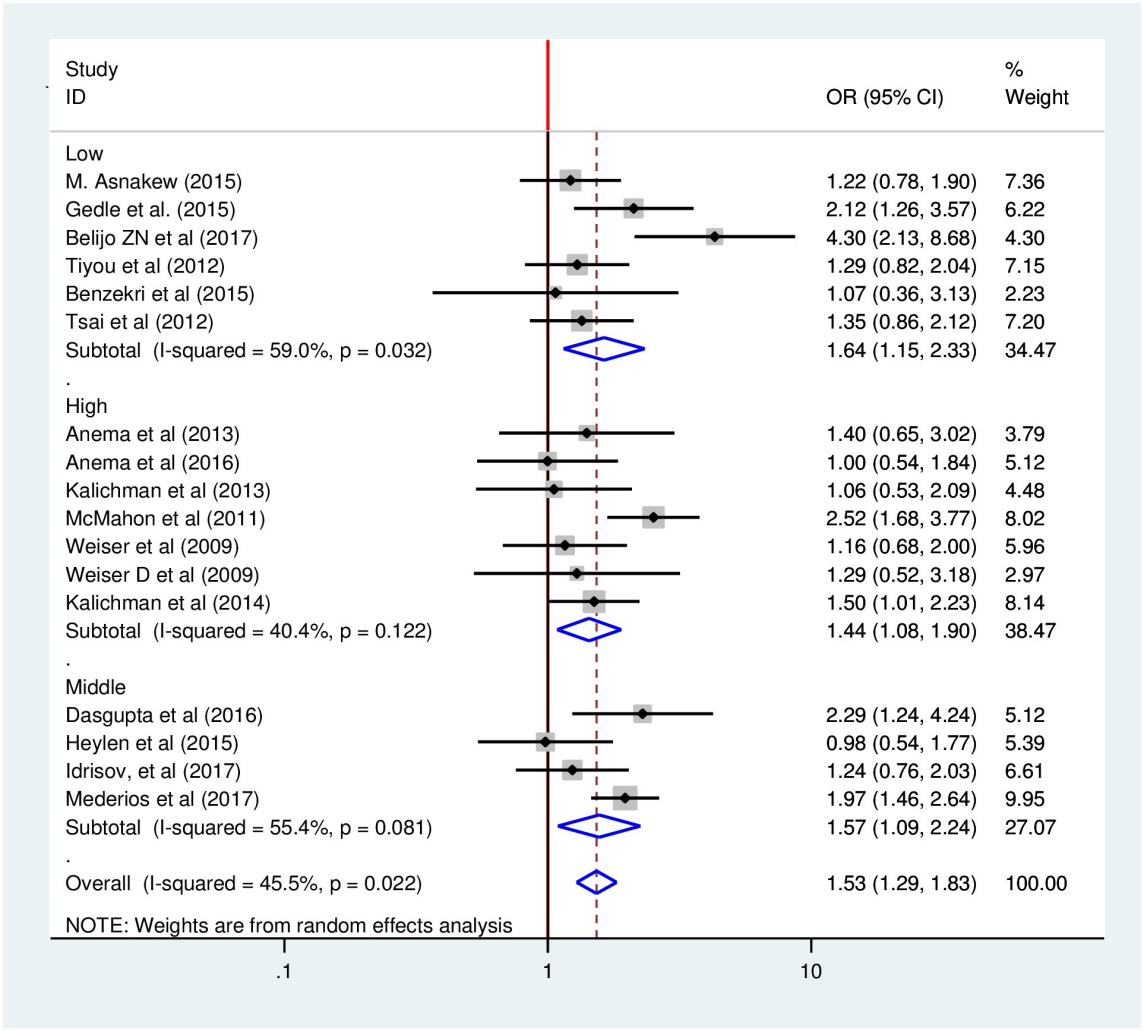
Sub-group analysis of effect of gender on food insecurity among HIV-infected adults receiving antiretroviral therapy by country income level from 2009–2017.

In addition, we performed sub-group analysis by country income level to minimize the potential random variations between studies by comparing the effect of gender on food insecurity HIV-infected adults. The analysis indicated that studies conducted in high-income countries found weaker associations than those in low and middle-income countries. The odds of developing food insecurity among female HIV-infected adults was 44% higher compared to male HIV-infected adults in the studies conducted in high-income countries, with an odds ratio of 1.44 (95% CI: 1.08, 1.90). While the odds of developing food insecurity among female HIV-infected adults was 64% and 57% higher compared to male HIV-infected adults in the studies conducted in low-and middle-income countries with odds ratios of 1.64 (95% CI: 1.15, 2.33) and 1.57 (95% CI: 1.09, 2.24) respectively (Fig 6).

## Discussion

Our review and meta-analysis estimated the pooled effect of gender on food insecurity among HIV-infected adults receiving anti-retroviral therapy. The review and analysis demonstrated that the gender of HIV-infected adults has strong statistically significant effect on food insecurity. The odds of developing food insecurity among female HIV-infected adults receiving anti-retroviral therapy were higher than male HIV infected adults.

The significant effect of gender on food insecurity found in the current systematic review and analysis is in line with the findings of a global gap analysis systematic review on food insecurity and HIV/AIDS in which significant inequity in the experience of food insecurity by gender was found, with females being most at risk in both resource-rich and resource-limited settings[35]. This finding also consistent with the finding of a systematic review for formulating a conceptual framework for food insecurity and health in which female HIV-infected adults were most affected by food insecurity. [16]. As discussed above, these findings may be due to the fact that most females in the relationship have little power over household resource and food allocation, and that they serve as caregivers. which in turn, inhibits in their ability to make further investments in their own skills and education, increasing their susceptibility to food insecurity.

The finding of current systematic review was also consistent with the evidence of global policy review by the International Food Policy Research Institute which indicated gender inequity shapes power relations and risk[7]. The significant effect of gender on food insecurity in the current review is in line with the finding of narrative reviews and international food assistance program guidelines in which food insecurity remains a challenge for women across diverse settings[36, 37].

Our analysis indicated a relatively consistent significant effect of gender on food insecurity among HIV-infected adults across study designs and country income level. We found that cohort studies had weaker associations than cross-sectional studies due to the lack of temporal relationship assurance in cohort studies.

In addition, our analysis found that the weakest associations between food insecurity and gender occurred in the studies conducted in high-income countries and the strongest in low and middle income country settings. This most likely due to a relative lack female social development particularly a lack of education in low-and middle-income settings as well as lower female power in decision-making around resource allocation.

We used comprehensive search strategies in our review systematic review and meta-analysis. We searched both published and unpublished studies through different database searches. We used random effects model to address the issues of potential variability across studies. More than one assessor was used in the quality assessment and appraisal process using JBI-MAStARI. Nevertheless, the restriction of studies published in English language limited the number of studies included in meta-analysis.

## Conclusion

The systematic review and meta-analysis indicated a consistent, and statistically significant effect of gender on food insecurity among HIV-infected adults receiving anti-retroviral therapy. Being female was found to have a positive significant effect on the development of food insecurity across a range of settings; however, the association was strongest for low- and middle-income countries. The review found strong significant positive effect on the development of food insecurity among female HIV infected adults living in the low and middle income countries compared to female HIV infected adults living in high income countries. These findings suggest that policy makers, planners, and program managers in these settings should pay attention to gender dynamics in the design and implementation of HIV prevention and control program that address food insecurity. Food, nutrition and HIV intervention programs should be culture and context specific, to address the sex/gender related vulnerability to food insecurity of HIV-infected adults.

## Supporting information

S1 Table PRISMA 2009 checklist. (DOC)

S2 Table Critical appraisal BI-MAStARI instrument. (DOCX)

S1 Dataset Gender effect dataset. (DTA)

## Abbreviations

AIDS: Acquired Immunodeficiency Syndrome
HFIAS: Household Food Insecurity Access Scale
HIV: Human immune virus
WHO: World Health Organization
