# Supplementary material for: The effect of gender on food insecurity among HIV-infected people receiving anti-retroviral therapy: A systematic review and meta-analysis
Source: PLoS One. 2019 Jan 7;14(1):e0209903. doi: 10.1371/journal.pone.0209903 (PMC6322826; doi:10.1371/journal.pone.0209903)
Supplement: S2 Table — (DOCX) [file pone.0209903.s002.docx]

**Appendix 1: Appraising descriptive/case series studies JBI-MAStARI Instrument**


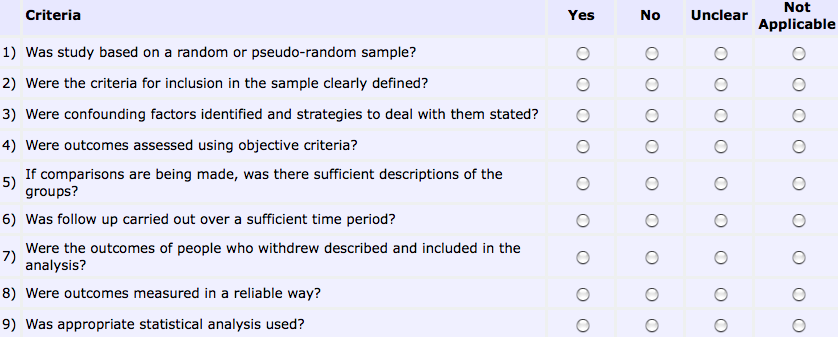


**Appraising comparable Cohort and Case-control studies JBI-MAStARI Instrument**

**
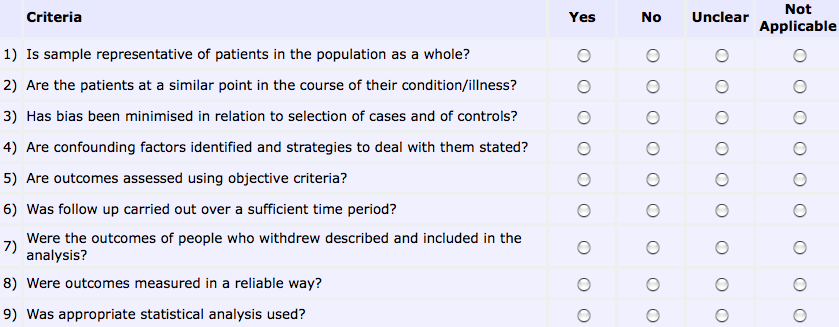
**
